# Biomimetic Multispiked Connecting Ti-Alloy Scaffold Prototype for Entirely-Cementless Resurfacing Arthroplasty Endoprostheses—Exemplary Results of Implantation of the Ca-P Surface-Modified Scaffold Prototypes in Animal Model and Osteoblast Culture Evaluation

**DOI:** 10.3390/ma9070532

**Published:** 2016-06-29

**Authors:** Ryszard Uklejewski, Piotr Rogala, Mariusz Winiecki, Renata Tokłowicz, Piotr Ruszkowski, Maria Wołuń-Cholewa

**Affiliations:** 1 Department of Medical Bioengineering Fundamentals, Institute of Technology, Casimir the Great University, Karola Chodkiewicza Street 30, Bydgoszcz 85-064, Poland; winiecki@ukw.edu.pl; 2 Department of Process Engineering, Institute of Technology and Chemical Engineering, Poznan University of Technology, Marii Sklodowskiej-Curie 2, Poznan 60-965, Poland; renata.toklowicz@wp.pl; 3 Department of Spine Surgery, Oncologic Orthopaedics and Traumatology, Poznan University of Medical Sciences, 28 Czerwca 1956 135/147, Poznan 61-545, Poland; gabinet.rogala@gmail.com; 4 Department of Pharmacology, Poznan University of Medical Sciences, Rokietnicka 5A, Poznan 60-806, Poland; pruszkowski@gmail.com; 5 Department of Cell Biology, Poznan University of Medical Sciences, Rokietnicka 5D, Poznan 60-806, Poland; doskon@ump.edu.pl

**Keywords:** multispiked connecting scaffold (MSC-Scaffold) Ti-alloy prototype, bone-implant biomimetic prototype interface, osteoinduction and osteointegration potential, resurfacing arthroplasty RA endoprostheses, animal model evaluation, osteoblast cell culture evaluation

## Abstract

We present here—designed, manufactured, and tested by our research team—the Ti-alloy prototype of the multispiked connecting scaffold (MSC-Scaffold) interfacing the components of resurfacing arthroplasty (RA) endoprostheses with bone. The spikes of the MSC-Scaffold prototype mimic the interdigitations of the articular subchondral bone, which is the natural biostructure interfacing the articular cartilage with the periarticular trabecular bone. To enhance the osteoinduction/osteointegration potential of the MSC-Scaffold, the attempts to modify its bone contacting surfaces by the process of electrochemical cathodic deposition of Ca-P was performed with further immersion of the MSC-Scaffold prototypes in SBF in order to transform the amorphous calcium-phosphate coating in hydroxyapatite-like (HA-like) coating. The pilot experimental study of biointegration of unmodified and Ca-P surface-modified MSC-Scaffold prototypes was conducted in an animal model (swine) and in osteoblast cell culture. On the basis of a microscope-histological method the biointegration was proven by the presence of trabeculae in the interspike spaces of the MSC-Scaffold prototype on longitudinal and cross-sections of bone-implant specimens. The percentage of trabeculae in the area between the spikes of specimen containing Ca-P surface modified scaffold prototype observed in microCT reconstructions of the explanted joints was visibly higher than in the case of unmodified MSC-Scaffold prototypes. Significantly higher Alkaline Phosphatase (ALP) activity and the cellular proliferation in the case of Ca-P-modified MSC-Scaffold pre-prototypes, in comparison with unmodified pre-prototypes, was found in osteoblast cell cultures. The obtained results of experimental implantation in an animal model and osteoblast cell culture evaluations of Ca-P surface-modified and non-modified biomimetic MSC-Scaffold prototypes for biomimetic entirely-cementless RA endoprostheses indicate the enhancement of the osteoinduction/osteointegration potential by the Ca-P surface modification of the Ti-alloy MSC-Scaffold prototype. Planned further research on the prototype of this biomimetic MSC-Scaffold for a new generation of RA endoprostheses is also given.

## 1. Introduction

Current standard fixation of the endoprostheses components used in total resurfacing hip arthroplasty (TRHA) in orthopedic surgery is the hybrid fixation: cemented short-stem femoral component fixation in periarticular cancellous bone and cementless acetabular component fixation due to bone-ingrowth into a porous coating of this component [1]. TRHA is offered for the young and active adults with advanced osteoarthritis (OA) as an alternative to conventional stemmed total hip arthroplasty (THA). Although, in case that the hybrid RHA cement provides immediate post-operative fixation of the femoral component, it is known that cemented implants gradually loosen from bone (due to cement damage),which has been highlighted, especially in young, active patients exposed to high shear stress forces [2]. The femoral fractures incidence correlated with the extent of osteonecrosis (ON) are reported to be resulting from cement fixation [3,4]. Therefore, many authors indicate that it is logical to achieve a cementless fixation on the femoral side of RHA [5,6,7,8] in order to avoid post-operative problems with cement (like femoral head collapse, excessive cement penetration, fatigue failure, potential for thermal necrosis, etc.) and to accomplish long-term biological fixation of cementless femoral components of RHA [1].

Our biomimetic Ti-alloy prototype of the MSC-Scaffold is such an option and the essential innovation in an interfacing (fixation) technique of the RA endoprostheses components in the periarticular trabecular bone. It was designed, manufactured, and tested by our research team [9,10,11,12,13,14,15,16,17], basing on the concept of multispiked (needle-palisade) bone fixation, invented by one of the co-authors [18,19,20].

The purpose of this article is to present the current state and the perspectives of our research on the MSC-Scaffold Ti-alloy prototype for RA endoprostheses, and to show the unpublished exemplary results of the performed pilot experimental study on this MSC-Scaffold prototype in animal models (according to the ISO 10993 requirements) and the osteoblast culture evaluation—to demonstrate the effect of the enhancement of the osteoinduction/osteointegration potential of the Ca-P surface-modified MSC-Scaffold prototypes (some of these results were shown in oral presentation at the European Society of Biomaterials Conference ESB 2015 [21]). The current state of our research is a part of the wider basic bioengineering research program leading to establishing the suitable constructional directives for the engineering design and manufacturing of the prototypes of partial and total RA endoprostheses with biomimetic MSC-Scaffold fixation techniques for knee and hip joints, which—according to ISO 10993 recommendations—will be verified in the so-called full standard experimental study in animal models in the planned next research project. On the basis of these verification results in animals, it will be possible to carry out the first experimental surgical RA endoprosthesoplasty in humans with the use of our biomimetic RA endoprosthesis prototype.

## 2. Current State of Research on the MSC-Scaffold Prototype for Biomimetic RA Endoprostheses

The spikes of the MSC-Scaffold prototype mimic the interdigitations of the articular subchondral bone, which is the natural biostructure interfacing the articular cartilage with the periarticular trabecular bone [22,23,24]. The interspike space in the MSC-Scaffold prototype maintains the space for bone regeneration, i.e., it constitutes the complementary interfacing (osteoconductive) region for interpenetration of the periarticular trabecular bone tissue in diarthrodial human joints treated with such RA endoprostheses. This new RA endoprostheses fixation technique is prominent with the biomimetism of its MSC-Scaffold: (1) respecting the microstructure of the periarticular subchondral and cancellous bone tissue; (2) preserving the posterolateral and medial epiphyseal femoral arteries (subcapsular arteriae retinaculares: superior and inferior) of the femoral head; as well as (3) providing the close-to-natural load transfer with similar mechanical properties to bone, which—as in the biomechanical environment of a natural hip joint—goes through the bone trabeculaes in the head and the neck of the femur, and then along the femoral shaft.

Against many observed failures of the standard fixation technique of contemporary metal-on-metal THRA endoprostheses, where the femoral component is fixed with the use of cement, our prototype of biomimetic MSC-Scaffold can be regarded a promising breakthrough in bone-implant advanced interfacing in joint RA endoprostheses fixation techniques. Our advanced biomimetic Ti-alloy prototype interfacing with bone (i.e., the MSC-Scaffold prototype) manufactured with modern advanced laser additive technology, opens a new generation for the first biomimetic RA endoprostheses applicable in most diarthrodial joint arthroplasties (hip, knee, shoulder, elbow, etc.) used in orthopedic surgery treatment.

As a part of our recent research on establishing the constructional directives for biomedical engineering design of prototypes of partial and total RA endoprostheses of the knee and hip joints with the MSC-Scaffold, the structural analysis and the functionalization of the MSC-Scaffold prototype was performed, with respect to the expected requirements of its pro-osteoconduction functionality and its proper successive osteointegration with the periarticular trabecular bone tissue [25]. The major purpose of this research [25] was the examination of the manufactured in selective laser melting (SLM) technology MSC-Scaffold prototypes and the evaluation of the possibilities to affect its interspike structural pro-osteoconductive potential. Awareness of the appearing technological limitations of the SLM technology has allowed for the necessary modification in the CAD model design of the MSC-Scaffold, taking into account the adjustments of the technological limitations.

The pilot implantation study in animals was structurally functionalized in [25] the MSC-Scaffold interfacing system prototype, and has brought us promising results [26]. The histopathological evaluation of the bone tissue surrounding the spikes of MSC-Scaffold pre-prototypes implanted in experimental animal model (swine) and the results obtained after 10-day culture of human osteoblasts on these pre-prototypes, have allowed us to conclude that the scaffolding effect was obtained with our MSC-Scaffold pre-prototypes. The majority of its interspike pore space was filled by newly-formed and properly-remodeled bone tissue, providing primary biological fixation of the MSC-Scaffold pre-prototypes in periarticular cancellous bone. Some histopathological findings in the periscaffold domain, like fibrous connective tissue and metallic particles near the MSC-Scaffold spikes’ base edges, prompted us to consider the necessity of optimizing the design the MSC-Scaffold in the regions of its interspike space near the spikes base edges to provide more room for new bone formation in this region. The histopathological evaluation of the periscaffold spikes’ bone tissue has also let us conclude that, to enhance the osteoinduction/osteointegration potential of the MSC-Scaffold, its spikes’ surface should be physicochemically modified, for example, with hydroxyapatite.

Attempts to modify the bone contacting surfaces of the MSC-Scaffold prototype by the method of electrochemical cathodic deposition (ECD) of calcium phosphates has been undertaken initially at constant current densities with subsequent immersion of the MSC-Scaffold pre-prototype in solution of simulated body fluid (SBF) in order to transform the amorphous Ca-P coating into HA-like coating [27]. The obtained results have shown that ECD allows achieving a HA-like coating in short time. It was observed that it is possible to control the deposition of Ca-Ps on surfaces of our MSC-Scaffold prototypes by means of adjusted current density. HA-like crystal forms were observed in the modified coating and the energy dispersive spectroscopy (EDS) of the modified MSC-Scaffold surface has shown that the coatings were characterized by the Ca/P ratio equal to about 1.67—which corresponds with the value of native bone HA.

Our further research on the Ca-P surface modification processes of the MSC-Scaffold pre-prototypes in galvanostatic ECD_j=const_ conditions vs. potentiostatic ECD_V=const_ conditions has shown the higher repeatability of results in case of the potentiostatic process ECD_V=const_. For this reason, the essential research of the effect of the process parameters of Ca-P ECD on the modified surface properties of our pre-prototypes was carried out potentiostatically [28]. According to the conclusions derived from our research presented in [26], design of the MSC-Scaffold in the regions of its interspike space near the spikes’ base edges has been changed. For the research on the effect of the process parameters of Ca-P ECD_V=const_ on the modified surface properties of the MSC-Scaffold, the two new pre-prototype design variants, varying with the distance between the spikes’ bases: 200 μm (PSc_200_) and 350 μm (PSc_350_), both circumferentially and radially, have been used. The negative result of the Ca-P ECD_V=const_ surface modification of the PSc_200_ MSC-Scaffold pre-prototypes and the effect of blocking of the interspike space of those MSC-Scaffold pre-prototypes by the Ca-P deposits has led to deciding to abandon further research with the use of this geometrical variant of the MSC-Scaffold pre-prototype. The detailed presentation of the results of our investigation on the process of combined ECD of Ca-P on bone contacting surfaces of the MSC-Scaffold pre-prototypes is subject of a separate paper [29].

## 3. Materials and Methods

### 3.1. Manufacturing of the Ti-Alloy MSC-Scaffold Pre-Prototypes

**Figure 1:**
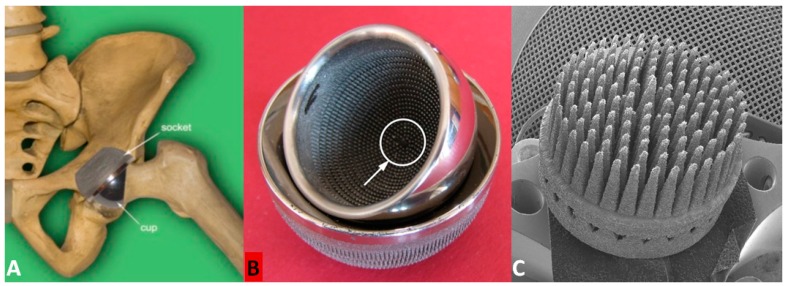

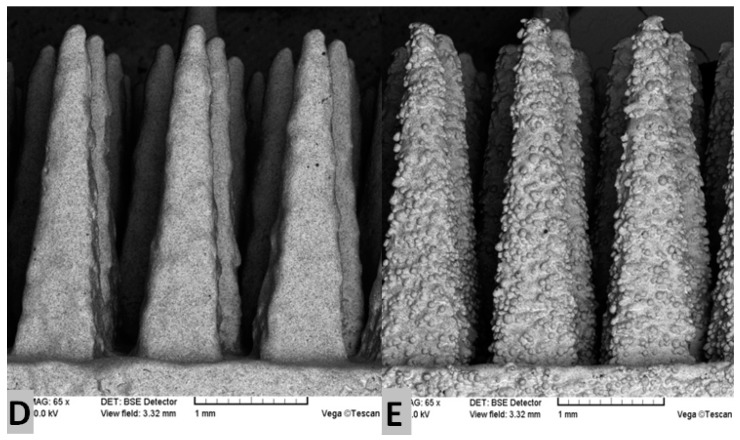
(**A**,**B**) Prototype of the biomimetic entirely-cementless total resurfacing hip arthroplasty (TRHA) endoprothesis with the multispiked connecting scaffold (MSC-Scaffold) manufactured with Selective Laser Technology (SLM); (**C**) the exemplary pre-prototype of the MSC-Scaffold directly after the SLM manufacturing process; (**D**) the SEM photograph showing numerous micro residues in the form of unmelted powder particles and sphere-like shapes on the multi-spiked surface of the scaffold; and (**E**) the spikes of the MSC-Scaffold after the manual post-production blasting treatment of its multi-spiked surface with use of an experimentally-customized abrasive mixture.

The research was carried out on the MSC-Scaffold pre-prototypes designed as fragments of the central part of the total hip resurfacing arthroplasty (THRA) endoprosthesis femoral component. The prototype of the THRA endoprosthesis with the MSC-Scaffold manufactured with SLM technology of Ti4Al6V powder is presented in Figure 1A,B. The CAD model of the MSC-Scaffold pre-prototypes were designed for the purpose of this research in a geometrical configuration variant characterized by the distance between the spikes’ bases of 350 μm (PSc_350_), both circumferentially and radially. The MSC-Scaffold pre-prototypes were manufactured in the REALIZER II 250 SLM machine (MTT Technologies Group, Lübeck, Germany) and the manufacturing was subcontracted to the Centre of New Materials and Technologies at West Pomeranian University of Technology, Szczecin, Poland. The pre-prototypes were manufactured of Ti6Al4V powder (grain size distribution from 5 to 50 µm; the mean grain size—35 µm).The process parameters applied during the SLM manufacturing of the prototypes were laser: 100 watt, layer thickness: 30 µm, laser spot size: 0.2 mm, scan speed: 0.4 m/s, and laser energy density: 70 J/mm^3^. In Figure 1C the exemplary pre-prototype of the MSC-Scaffold directly after the SLM manufacturing process is shown. 

The process of additive layer manufacturing with the SLM method leaves, allover the multi-spiked surface of the scaffold, many adjacent micro sphere-like residues of not-entirely-melted particles of Ti6Al4V metallic powder. The manual post-production blasting treatment of the multi-spiked surface was carried out with the use of an experimentally-customized abrasive mixture composed in equal proportions of white aloxite F220 (~53–75 μm), white aloxite F320 (~29.2 μm ± 1.5%), and glass microbead blasting (~30 μm ± 10%) [30]. The SEM photograph showing numerous micro residues in the form of unmelted powder particles and sphere-like shapes on the multi-spiked surface of the scaffold is presented in Figure 1D, while at the SEM photograph presented in Figure 1E the spikes of the MSC-Scaffold after the manual post-production blasting treatment of its multi-spiked surface are shown.

### 3.2. Ca-P Surface Modification of the Ti-Alloy MSC-Scaffold

Before the Ca-P surface modification process, the pre-prototypes of the MSC-Scaffold were cleaned chemically, respectively, in distilled water, ethanol, acetone, and then three times in distilled water; each stage took 15 min. Afterwards, the acid-alkaline preprocessing treatment was applied to the MSC-Scaffold pre-prototypes in the sulfuric acid (H_2_SO_4_) for 40 min at temperature of 60 °C and, subsequently, in the sodium hydroxide (NaOH) for 40 min at 80 °C. Then, the MSC-Scaffold pre-prototypes were modified with an ECD process in the solution containing 0.042 M Ca(NO_3_)_2_ and 0.025 M NH_4_H_2_PO_4_, at room temperature, for 1 h. The ECD process was carried out at constant current density = 5 mA/cm^2^, a gold electrode was used as the anode, and the source of electricity was a potentiostat/galvanostat Metrohm Autolab PGSTAT 302N. In order to transform the deposited amorphous coating of calcium and phosphate into an HA-like coating, the MSC-Scaffold prototypes were incubated at 37 °C, while the temperature was maintained by means of a thermostat for 24 h in a solution of SBF of the following composition: 6.8 g/L NaCl, 0.4 g/L KCl, 0.2 g/L CaCl_2_, 0.2048 g/L MgSO_4_∙7H_2_O, 0.1438 g/L NaH_2_PO_4_∙H_2_O, and 1.0 g/L NaHCO_3_. The qualitative SEM analysis and the EDS microanalysis of the chemical composition of the modified microstructure of the MSC-Scaffold pre-prototype were made using a Hitachi TM-3030 scanning electron microscope (Hitachi High-Tech Technologies Europe GmbH, Krefeld, Germany) equipped with an EDS system (Oxford Instruments, Oxfordshire, UK) and Vega 5135 (Tescan, Brno-Kohoutovice, Czech Republic) equipped with an EDS system (Princeton Gamma-Tech, Inc., Princeton, NJ, USA). The surface mapping on three randomly-selected subareas of spikes’ modified lateral surface of each MSC-Scaffold pre-prototype was performed using a specialized software analyzer enclosed in an EDS system. On the basis of the mapping, the regions with Ca-P deposited on the spikes’ lateral surface were indicated and the average degree of lateral surface of the spikes’ coverage was determined. In each of analyzed subareas the 10 pointwise measurements of the chemical composition were made and the Ca/P ratios were calculated. The analyses of the degree of lateral surface of the spikes’ coverage and the uniformity of the deposited coating were made using the professional software tool ImageJ (National Institutes of Health, Bethesda, MD, USA).

**Figure 2:**
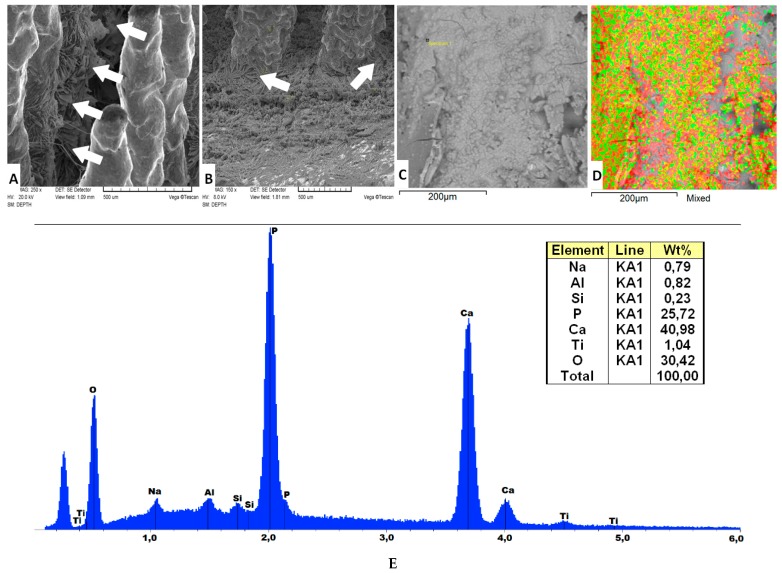
(**A**,**B**) SEM images of the MSC-Scaffold pre-prototypes subjected to electrochemical modification at a current density 5 mA/cm^2^ followed by 24 h immersion in SBF, the arrows show the plate-like shaped hydroxyapatite-like crystals on the lateral at the lateral surface of the MSC-Scaffold’s spikes (**A**), as well as at the base of the scaffold near the spikes’ edges (**B**); (**C**,**D**) the exemplary region on the spikes’ lateral surface and the photograph showing the same region with mapping performed using a specialized software analyzer enclosed in an EDS system; the red color corresponds to atoms of calcium, and green and light-green, to phosphorus atoms; and (**E**) the exemplary EDS spectrogram dealing with a region on the spikes’ lateral surface, and the corresponding chemical composition showing that the Ca/P ratio is equal to 1.59.

The SEM images in Figure 2A,B present the MSC-Scaffold pre-prototypes subjected to electrochemical modification at current density 5 mA/cm^2^ followed by 24 h immersion in SBF. The arrows show the plate-like shaped hydroxyapatite-like crystals on the lateral surface of the MSC-Scaffold’s spikes, as well as at the base of the scaffold near the spikes’ edges. In Figure 2C the exemplary region on the spikes’ lateral surface correspond to the photograph in Figure 2D showing the same region with mapping performed using a specialized software analyzer enclosed in an EDS system; the red color corresponds to atoms of calcium, and green and light-green, to phosphorus atoms. In Figure 2E, the exemplary EDS spectrogram dealing with a region on the spikes’ lateral surface is presented.

According to the EDS spectrum shown in Figure 2E, the main elements detected on the lateral surface of the MSC-Scaffold’s spikes were: Ca, P, and O, indicating that Ca-P was present in the coating deposition. The Ca/P ratio evaluated on the base of the EDS spectra is equal to 1.59, which means (see e.g., [31]) that an HA-like coating was produced on the lateral surface of spikes of the MSC-Scaffold. The Ti, Al, and V elements found in the EDS analysis may originate from the Ti6Al4V substrate because, as it can be seen in Figure 2D, the slight fragments of the lateral surface of the MSC-Scaffold’s spikes were not Ca-P coated, i.e., the Ca-P coating is not uniform. The Si elements and, in part, the Al elements showed in the EDS spectra may come from the abrasive mixture applied for the blast cleaning of surface of the MSC-Scaffold’s spikes, which consists mostly of aloxite (Al_2_O_3_) and glass microbeads (70% of SiO_2_).

The Ca/P ratios found (1.56 ÷ 1.74) on the lateral surface of spikes of the MSC-Scaffold pre-prototypes correspond with the values of native hydroxyapatite [26] of bone and, as a result of the performed modification, the quite uniform HA-like coating was produced with single micro-cracks. More results of the research on the Ca-P surface modification process were presented in [26,27] and are the subject of a separate paper [29].

### 3.3. Implantation in an Animal Model of Pre-Prototypes of MSC-Scaffolds for RA Endoprostheses

Research in animals (swine, White Polish Large breed) were carried out to test the MSC-Scaffold pre-prototype surfaces modified with Ca-Ps and unmodified surfaces. Four MSC-Scaffold pre-prototypes in total were implanted (in veterinary clinic conditions) into the knee joint of two experimental animals. The biointegration with bone tissue after eight weeks from implantation was evaluated. During the surgical implantation of the MSC-Scaffold pre-prototypes the general anesthesia by inhalation with endotracheal intubation and anesthetic monitoring were applied, given Cepetor 0.01–0.04 mg/kg BW, intravenously; anesthesia was maintained with inhaler Isoflurane (Forane) controlled by pulse oximetry with a heart monitor; Dräger AT-: premedication, once given intramuscularly (in the same syringe) Cepetor/Medetomidine hydrochloride/(0.02–0.04 mg/kg BW i.m.) and Lewometadon (0.25–0.5 mg/kg BW i.m.). General anesthesia was induced using ketamine hydrochloride (30 mg/kg i.v.). Metamizole sodium (30 mg/kg i.v.) and infusion fluids were administered.

A medial parapatellar surgical approach to the swine knee joint was applied. An anteromedial slim incision of ca. 20 cm length over the operated right knee joint was done. Approach to the knee joint between the lateral margin of patella and the external side of patellar ligament and then between vastus lateralis muscle and rectus femoris muscle was applied. The articular capsule was opened on the lateral side of patella, and then the patella was dislocated medially. Hemostasis was done. Patellofemoral region of the knee joint was exposed. The implantation sites in both femoral condyles were prepared using a surgical drill. Subchondral bone holes were gradually widened until the final size with the milling cutter to harbor the implant. During drilling and milling processes the bone holes were continuously cooled with saline. The holes in condyles were irrigated with saline and bone debris was removed. The two pre-prototypes (i.e., Ca-P modified and non-modified) of the MSC-Scaffold were inserted under the articular cartilage surface of the medial and lateral femoral condyli of each swine knee joint. Implant insertion into the bone holes in femoral condyli was performed using surgical impact. Reposition of the patella on the anatomical site was made. A layered suture of the wound was applied. An antibiotic regime after implantation was introduced: penicillin powder was given into the subcutaneous layer at the end of surgery, the wound was covered by a mesh impregnated with penicillin, and antiseptic dressing was applied. After the surgery Amikacin (Biodacyna) 1 g twice a day i.v. (or i.m.) for three days was given. On the third day after the surgery the swine were allowed full weight bearing.

### 3.4. Harvesting of Joints Containing Implants

Eight weeks after implantation, the explantation (i.e., surgical harvesting of operated joints with implants) was performed in the veterinary department operating room (premedication and general anesthesia as by implantation), and the two knee joints with the MSC-Scaffold pre-prototypes were harvested from animals. The procedure was finished by euthanasia of the animals (Morbitan/Pentobarbital natrium/in lethal doses 200 mg/kg BW i.v., according to the protocol approved by the local animal ethics committee. The explanted knee joints were subject of radiological examinations using the 2D Digital Specimen Radiography System (XPERT 40, Kubtec, Milford, CT, USA).

### 3.5. Micro-CT Reconstructions and Examination

The micro-CT examination of the explanted knee joint was conducted using a high-energy micro-CT scanner (SkyScan 1173 microtomograph, Bruker, Kontich, Belgium). The specimens immersed in formalin were mounted on a rotary stage and scanned in their entirety. The scanning parameters were: source voltage 130 keV, source current: 61 μA, resolution: 9.92 μm, filter: 0.25 mm brass, exposure time: 4000 ms, rotation: 3600, every 0.2°, scanning time: about 6 h. 3D visualization and 2D image analysis of micro-CT reconstructed knee joints with the MSC-Scaffold was performed using a SkyScan CT-analyser. 3D images micro-CT reconstructed areas of bone-implant specimens were identified and distinguished on the basis of radiological density as Ti-Alloy MSC-Scaffold, trabeculae, and soft tissues, including bone marrow. On the eight reference levels of area between the spikes perpendicularly intersecting axes of the MSC-Scaffold below the tops and spaced 0.5 mm from each other, the trabeculae share in specimens containing, and non-containing, Ca-P-modified MSC-Scaffold was compared.

### 3.6. Preparation of the Samples and Histological Examination

Following explantation, the specimens were fixed in 10% phosphate-buffered formalin, pH 7.25, for seven days and dehydrated in serial concentrations of ethanol (70%, 80%, 90%, 99%, 100%, and 100% *v*/*v*) for two days at each concentration, and embedded in resin. Thick sections (200 µm) were cut using a rotating wheel saw (IsoMet 4000 Linear Precision Saw, Buehler, Düsseldorf, Germany) and manually ground to a thickness of 20 µm using the MetaServ 250 Grinder-Polisher (Buehler, Düsseldorf, Germany). Sections were stained with hematoxylin-eosin and examined by light microscopy (Olympus CX41, Olympus, Tokyo, Japan).

### 3.7. Osteoblast Cell Biological Tests (Including Alkaline Phosphatase Assay)

Biological tests of the MSC-Scaffold prototypes (Ca-P surface modified and non-modified) in osteoblast cell culture were conducted where cell proliferation around the spikes and enzymatic activity of alkalic phosphatase were evaluated.

#### 3.7.1. Cell Proliferation Test

Human osteoblasts (U-2 OS cell line) were purchased from the American Type Cell Culture (ATCC, Manassas, MA, USA) and cultured in McCoy’s 5a Medium Modified (Sigma-Aldrich, St. Louis, MO, USA) supplemented with 10% fetal calf serum (FCS; Sigma, St. Louis, MO, USA), 2 mmol/L l-glutamine (Cambrex, Charles City, IA, USA), 100 IU/mL penicillin and streptomycin solution (Sigma, St. Louis, MO, USA) in a 5% CO_2_-humidified atmosphere at 37 °C. 2 × 10^4^ cells per unmodified and Ca-P-functionalized PSc_350_ pre-prototype of the MSC-scaffold were seeded and placed in 12-well culture plates and incubated up to five days in growing media in a humidified atmosphere (37 °C, 5% CO_2_) to allow cell attachment. Then, U-2 OS cells were stained with 0.1 μg/mL of Hoechst 33,342 and 0.125 μg /mL of propidium iodide (Sigma-Aldrich, St. Louis, MO, USA). The presence of intact, apoptotic, and/or necrotic cells was evaluated using a Zeiss LSM 780 confocal microscope (Jena, Germany). Signals were excited at 345 and 543 nm and fluorescence emission were selected with 460 and 560 nm bandpass filters.

#### 3.7.2. ALP Test

A human bone cell line (MG63) was provided by ATCC (USA) and cultured in Eagle’s Minimum Essential Medium (EMEM, Sigma-Aldrich, St. Louis, MO, USA) with addition of 10% fetal bovine serum (FBS, Sigma-Aldrich, St. Louis, MO, USA) and 1% of streptomycin and penicillin solutions and grown for 10 days. Then, the activity of alkalic phosphatase (ALP) was measured with the use of colorimetric test (Alkaline Phosphatase Assay Kit, Abcam, Cambridge, UK). First, the EMEM medium was removed from the culture and washed cells (1 × 10^5^) were homogenized in the assay buffer and centrifuged for 3 min. Then, all of the samples of the pre-prototype of the MSC-scaffold were placed in a 48-well microplate with the addition of 80 µL of the assay buffer. After that 50 µL of the 5 mM p-nitrophenyl phosphate (*pNPP*) as enzyme substrate solution were added to each well containing test samples as well as background control probes. Plates were then mixed in the orbital shaker for 5 min and incubated for 60 min at 25 °C protected from day light. Ten microliters of the ALP enzyme solution were added to each well containing *pNPP* standard and mixed. The main reaction between enzyme and the substrate was stopped by the addition of 20 µL of stop solution to each test and standard containing well. The whole plate was gently shaken and then ALP activity was measured at a wavelength of 405 nm in a microplate colorimetric reader Tecan (Tecan Group Ltd., Männedorf, Switzerland).

## 4. Results and Discussion

**Figure 3:**
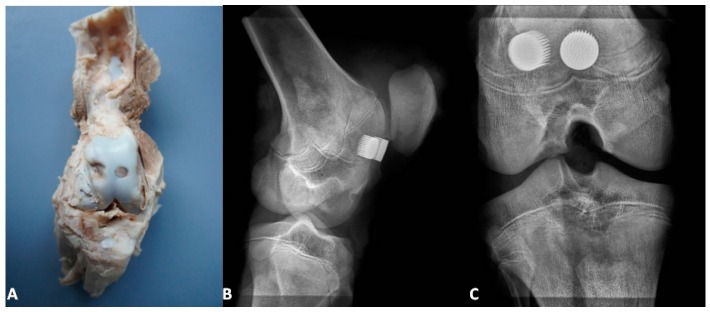
(**A**) The specimen with the implanted two pre-prototypes of the MSC-Scaffold explanted surgically at eight weeks after the implantation; the lateral (**B**) and anteroposterior (**C**) 2D digital X-ray radiograms of the explanted swine knee joint.

**Figure 4:**
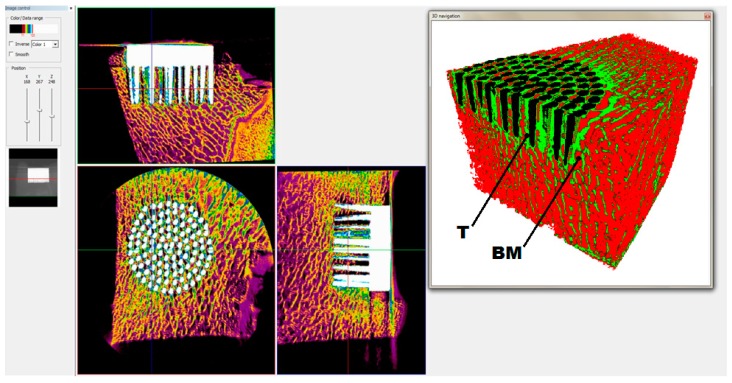
View of the window showing 3D micro-CT reconstruction of bone-implant specimen with sections in three dimensions and the exemplary 3D view of the MSC-Scaffold with unmodified spikes’ surface with distinction of the given elements, such as implant (black), trabeculae (T), and bone marrow (BM).

**Figure 5:**
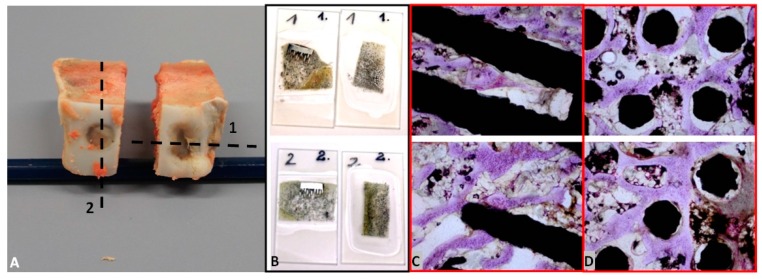
(**A**) The bone-implant specimens with the unmodified (1) surface and the Ca-P-modified (2) surface of the MSC-Scaffold pre-prototypes cut from the harvested knee joint; (**B**) the exemplary thin sections and the exemplary histopathological documentation of bone-implant specimens made on these bone-implant thin sections in longitudinal (**C**) and crosswise directions (**D**) to the axes of spikes (presented near to particular thin sections of unmodified surface and Ca-P-modified).

Figure 3, Figure 4 and Figure 5 present the exemplary results of the experimental study of biointegration of unmodified and Ca-P surface-modified PSc_350_ MSC-Scaffold prototypes conducted in an animal model. In Figure 3A the specimen of the operated swine knee joint with the implanted two preprototypes of the MSC-Scaffold resected surgically at eightweeks after the implantation is presented. In the surgically-explanted knee joint no femoral condyle destruction was observed. the two implanted pre-prototypes of the MSC-Scaffold showed good fixation without any signs of loosening. In Figure 3B,C the 2D digital X-ray radiograms (XPERT 40, Kubtec, Milford, CT, USA), respectively, lateral and anteroposterior, of the resected swine knee joint are shown. The radiological examination confirmed very good stability of the knee joint and no implant migration, as well as showed the spaces between spikes of the MSC-Scaffold of the both implanted MSC-Scaffold pre-prototypes filled by bone tissue.

Figure 4 presents the window of the CTanalyzer software showing the exemplary sections in three dimensions of the 3D micro-CT reconstruction of the explanted knee joint with the MSC-Scaffold. Figure 4 also presents the exemplary 3D view of bone-implant specimen with distinction performed on the basis of the radiological density of each element, such as the Ti-Alloy MSC-Scaffold (marked inblack), trabeculae (marked with the letter T and in red) and bone marrow (marked with letters BM and in green).

In Figure 5A, the bone-implant specimens with the unmodified (1) surface and the Ca-P-modified (2) surface of the MSC-Scaffold pre-prototypes cut from the harvested at eight weeks after the implantation knee joint are presented, together with the exemplary thin sections (Figure 5B) of bone with the unmodified (1) surface and the Ca-P-modified (2) surface mounted on a glass microscope slide before staining. In Figure 5C,D the exemplary eighth week after surgery histological (H + E) sections in, respectively, longitudinal and crosswise directions to the axes of spikes are presented.

The histological sections (Figure 5C,D) showed the trabecular bone tissue (violet color) between the spikes of the MSC-Scaffold and the soft tissue (connective tissue and bone marrow; white color). Some artifacts in the form of metal particles inserted into the interspike tissue area are also visible, resulting from the grinding process of the sections. The histological sections do not allow for the reliable assessment of the differences between the osteointegration of the periarticular bone tissue with the surfaces of the unmodified and Ca-P modified MSC-Scaffold pre-prototypes. For this reason, the quantitative evaluation of the osteointegration of these MSC-Scaffold pre-prototypes was performed by means of the percentage analyses of the trabeculae of cancellous bone tissue in the area between the spikes with use of the CTanalyzer software, on the micro-CT reconstructions of bone-implant specimens. The percentage values of trabeculae, in the area between the spikes on the given eight levels perpendicularly intersecting axes of MSC-Scaffold’s spikes below their tops and spaced 0.5 mm from each other of bone specimens containing the MSC-Scaffold pre-prototype with unmodified surface and the Ca-P-modified surface, are given in Table 1.

On the basis of the results shown in Table 1 we can clearly state the higher percentage of bone trabeculae in the area between the spikes of the scaffold prototype in the specimens containing the Ca-P surface-modified MSC-Scaffold prototype. The mean increase of percentage of bone trabeculae between the spikes due to the Ca-P modification of spikes surface is 12% ± 2% 2013—and it proves the possibility of enhancement of the osteoinduction/osteointegration potential of Ti-alloy MSC-Scaffold prototype by means of the Ca-P modification of bone-contacting surfaces of the scaffold.

**Figure 6:**
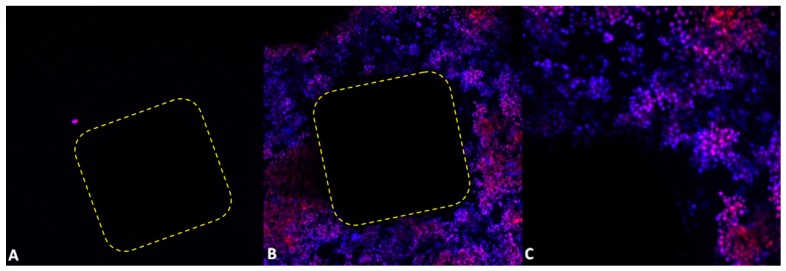
Fluorescent micrographs of five-day osteoblast culture after Hoechst 33342 and propidium iodide staining: (**A**) the unmodified and (**B**) the Ca-P-modified PSc_350_ pre-prototypes of the MSC-Scaffold (10× magnification); and (**C**) the PSc_350_ pre-prototype of the MSC-Scaffold (20× magnification); the dashed line indicates the contour of a spike base of the tested MSC-Scaffold pre-prototypes as seen from spike top.

Figure 6 shows results of five-day osteoblast culture on the unmodified surface and the Ca-P-modified PSc_350_ MSC-Scaffold prototype. Numerous proliferating osteoblast cells adjacent to the Ca-P-modified PSc_350_ variant of the MSC-Scaffold can be seen (blue cells); dead cells take a red color.

As shown in Figure 6, the unmodified PSc_350_ pre-prototype of the MSC-Scaffold’s surface showed a low number attached cells (Figure 6A). On the Ca-P-modified pre-prototypes of the MSC-Scaffold’s surface, cells were clustered around the spike, forming a large cellular network (Figure 6B). Using a higher magnification revealed that a great number of single cells attached to the surface were intact (blue color, Figure 6C), without signs of DNA fragmentation (no apoptotic cells were detected). However, necrotic cells were also observed (red color, Figure 6C).

**Figure 7:**
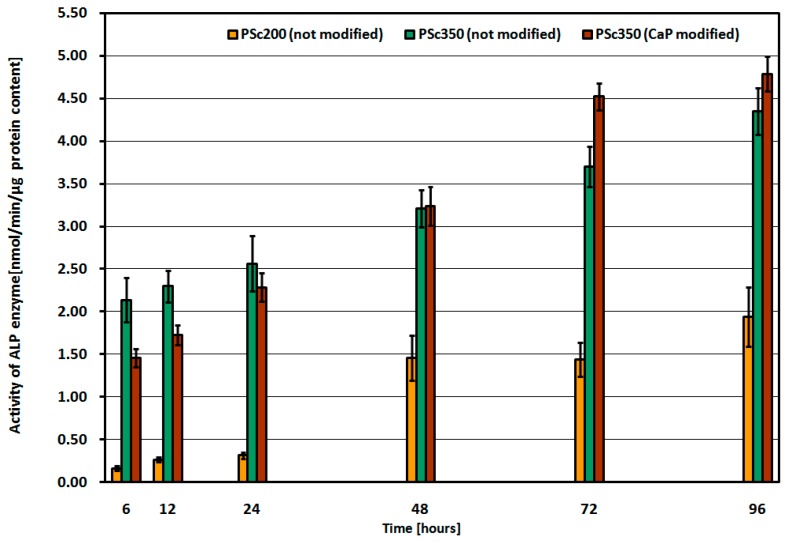
ALP enzyme activity in time of incubation in human bone cell line culture, and depending on examined MSC-Scaffold pre-prototype construction variant: PSc_200_ with unmodified surface, PSc_350_ with unmodified surface, and with Ca-P-modified surface.

The diagram presented in Figure 7 shows the activity of ALP enzyme in the function of incubation time in human bone cell line culture and depending on the examined MSC-Scaffold construction variants: PSc_200_ with unmodified surface, and PSc_350_ with unmodified surface, and with Ca-P-modified surface of spikes.

For all of the samples (pre-prototypes) of the MSC-Scaffold tested in human bone cell line culture the measured ALP activity increases proportionally with the time of the sample’s incubation. The ALP activity values for the MSC-Scaffold pre-prototypes with densely-spaced spikes (ca. 200 μm, PSc_200_) are significantly lower than the ALP activity values for the PSc_350_ pre-prototypes with less densely (ca. 350 μm) spaced spikes. For the MSC-Scaffold pre-prototypes with Ca-P-modified surface the ALP activity increases more rapidly with time as compared to the pre-prototypes with non-modified surfaces, and after 48 h of incubation the ALP activity for pre-prototypes with Ca-P-modified surfaces clearly exceed the ALP activity for non-modified pre-prototypes. Furthermore, the Ca-P surface modification process of the pre-prototypes showed no cytotoxic effect against the MG63 cell line used in the study. It follows from the study that the factor having a major impact on the ALP activity (and hence on the mineralization) is the proper distance between the spikes of the MSC-Scaffold prototype. The Ca-P modification of the spikes’ surface of the MSC-Scaffold Ti-alloy prototype is beneficial for bone cell proliferation and ALP activity and, hence, for the mineralization.

The osteoblast cell biological tests performed on construction variants of the MSC-Scaffold pre-prototypes varying with the distance between spikes bases: 200 μm (PSc_200_) and 350 μm (PSc_350_), both circumferentially and radially, have confirmed the identified (in our previous research [26]) necessity to optimize the design the MSC-Scaffold in the regions of its interspike space near the spikes’ base edges, indispensable to provide more room for new bone formation in this region. 

The low ALP activity for the PSc_200_ MSC-Scaffold pre-prototypes observed in human bone cell line culture, together with the earlier negative result of the ECD_V=const_ Ca-P surface modification of the PSc_200_ MSC-Scaffold pre-prototypes [29], clearly disqualifies this constructional variant of the MSC-Scaffold pre-prototype form further research. Thus, it has been proved to be reasonable to increase the distance between spikes of the MSC-Scaffold prototype up to 350 μm, both circumferentially and radially.

According to [4] the different survival rates correspond to different RHA models available on the market, ranging from 97.1% (better) to 80.9% (worst) at five-year follow-up. Forthis reason, concerns have been raised over the safety of some of these RHA endoprostheses and the suitability of their use, leading, for example, to the withdrawal in 2010 of the ASR (DePuy Orthopaedics Inc., Warsaw, IN, USA) due to a high failure rate [4]. Femoral neck fracture is a common cause of early RHA failure, accounting for up to 35% of revisions [32,33] while aseptic loosening of either femoral or acetabular components is another common cause of failure in RHA [34,35]. Post-RHA osteonecrosis (ON), described mostly in failures attributable to periprosthetic fractures or suggested as a cause of the periprosthetic fracture [36], is discussed as the effect of cementation [37] or intraoperative extraosseous vascular injury [38]—the polymerization properties of cement cause serious thermal damage in RHA, leading to the occurrence of femoral head collapse [39,40]. Moreover, during the femoral head component of RHA implantation, large quantities of cement are pressurized into cancellous bone, producing a thick cement mantle [2].

ON reportedly occurs predominantly in early- and midterm hip failures and may relate to impaired blood supply to the femoral head or heat injury at the time of surgery [41]. According to Zustin et al. [41] who analyzed histologically a series of 123 retrieved specimens from patients with five variousresurfacing systems (ASR of DePuy Orthopaedics Inc., Birmingham Hip Resurfacing of Smith & Nephew, Cormet of Corin Group PLC, DUROM of Zimmer Inc. and ReCAPof Biomet Inc.) with preoperative diagnosis other than ON, ON was found in 88% of cases and associated with 60% of periprosthetic fractures. Eighty-five of 123 revision measured were performed for perisprosthetic fractures, 8% for acetabular loosening and the remaining 23% for other causes such as groin pain of femoral component. The 60% of those periprosthetic fractures had complete ON of bone tissue proximally from the fracture line and was defined as postnecrotic fractures. Histologally advanced ON in majority of specimens was found and ON appeared to be causative in 46% of all failures attributable to postnecrotic periprosthetic fractures and collapse.

## 5. Conclusions and Planned Further Research

In this paper we have reviewed the current state of our research on the biomimetic Ti-alloy prototype of the MSC-Scaffold interfacing the RA endoprostheses components with periarticular cancellous bone. This MSC-Scaffold prototype was designed, manufactured in SLM technology, and tested by our research team with two research grants from the Polish Ministry of Science; the concept of such multispiked (needle-palisade) bone fixation was invented by one of the co-authors (P.R.) and it has international patent protection. The MSC-Scaffold prototype mimics the interdigitations of the articular subchondral bone, which is the natural biostructure interfacing the articular cartilage with the periarticular trabecular bone. With this biomimetic MSC-Scaffold prototype the major leap forward in bone-implant advanced interfacing in RA endoprostheses fixation techniques was done—opening a new generation of the first ever biomimetic RA endoprostheses, which can be applied for most diarthrodial joint arthroplasties (hip, knee, shoulder, elbow, etc.) used in orthopedic surgery treatment.

We have also presented in this paper the unpublished, exemplary-promising, results of the pilot surgical implantations of the MSC-Scaffold Ti-alloy pre-prototypes to the knee joint of laboratory swine and the results ofa 10-day evaluation in human osteoblast culture of these MSC-Scaffold pre-prototypes. These results have showed:

(1) obtaining the scaffolding effect (the majority of the interspike pore space of the MSC-Scaffold was filled by newly-formed and properly-remodeled bone tissue, providing primary biological fixation of the MSC-Scaffold pre-prototypes in periarticular cancellous bone) and have suggested the necessity of improving the osteoinduction/osteointegration potential of the MSC-Scaffold Ti-alloy surface, e.g., by means of the physicochemical Ca-P modification;
(2) the visibly higher percentage (ca. 12%) of trabeculae in the area between the spikes of specimens containing the Ca-P surface-modified MSC-Scaffold prototype observed in microCT reconstructions of the explanted knee joints eight weeks after implantation was noted;
(3) on histological bone-implant sections of the MSC-Scaffold prototype the signs of biointegration with bone (i.e., the presence of bone trabeculae in the interspike spaces of the MSC-Scaffold prototypes and in contact with these spikes on longitudinal and cross-sections of bone-implant specimens—the evident scaffolding effect) in both the unmodified and the Ca-P surface-modified MSC-Scaffold prototypes were evidenced; and
(4) from the studies in human bone cell line cultures, it follows that the factor having a major impact on the ALP activity (and hence on the mineralization) is the proper distance between the spikes of the MSC-Scaffold prototype; the Ca-P modification of the spikes’ surface of the MSC-Scaffold Ti-alloy prototype is beneficial for bone cell proliferation and ALP activity and, hence, for the mineralization and for the osteoinduction/osteointegration potential of the MSC-Scaffold Ti-alloy prototype.

Our performed pilot experimental studies on the biomimetic MSC-Scaffold Ti-alloy prototype in animal models and in osteoblast cultures have allowed us to establish the constructional directives for engineering design and the SLM manufacturing of this MSC-Scaffold prototype for biomimetic RA endoprostheses. The performed pilot studies are part of the wider basic bioengineering research program leading to establishing the suitable constructional directives for the engineering design and manufacturing of the prototypes of partial and total RA endoprostheses with the biomimetic MSC-Scaffold fixation technique for knee and hip joints, which—according to ISO 10,993 recommendations—will be verified in the so-called full standard experimental study in animal models in the planned next research project. On the basis of these verification results in animals, it will be possible to carry out the first experimental surgical RA endoprosthesoplasty in humans with use of our RA endoprosthesis prototype with biomimetic MSC-Scaffold fixation in periarticular bone.

The encouraging results of the pilot study of the MSC-Scaffold prototype for RHA endoprostheses in an animal model and in osteoblast cultures, together with a growing number of research premises to abandon those charged with a high risk of complications, such as hybrid RHA with cemented femoral components, strengthens our conviction that the biomimetic fixation technique by means of the spikes of the metallic MSC-Scaffold prototype leads to new solutions of biomimetic RA endoprostheses, minimizing the risk of disadvantages and post-operative complications of the current generation of RHAs.

## Figures and Tables

**Table 1:** Percentage of trabeculae in the interspike space of the explanted bone-implant specimens with non-modified and Ca-P-modified spikes surface of MSC-Scaffold prototype. The eight levels are the eight planes perpendicularly intersecting axes of the MSC-Scaffold’s spikes below their tops and spaced 0.5 mm from each other as counted from the top to the base of the spikes.

Reference Level	Trabeculae (%) (Non-Modified)	Trabeculae (%) (Ca-P Modified)	The Relative Difference (%)
Level 1	61.99	68.66	10.8
Level 2	60.37	68.79	13.9
Level 3	61.41	66.56	8.4
Level 4	56.82	64.02	12.7
Level 5	55.51	62.32	12.3
Level 6	52.77	59.83	13.4
Level 7	50.97	57.41	12.6
Level 8	53.70	58.75	9.4
		Mean ± SD:	12 ± 2
